# The efficacy and safety of exercise for prevention of fall-related injuries in older people with different health conditions, and differing intervention protocols: a meta-analysis of randomized controlled trials

**DOI:** 10.1186/s12877-019-1359-9

**Published:** 2019-12-03

**Authors:** Renqing Zhao, Wenqian Bu, Xianghe Chen

**Affiliations:** College of Physical Education, Yangzhou University, 88 Daxue South Rd, Yangzhou, 225009 Jiangsu Province China

**Keywords:** Exercise, Injurious falls, Older people, Health status, Exercise protocols

## Abstract

**Background:**

Whether exercise prevents fall-related injuries in different health conditions and with different training protocols is still unclear. This study aimed to determine the effect of exercise on fall-related injuries by participant characteristics and divergent exercise protocols. The safety and compliance of exercise were also examined.

**Methods:**

Electronic database searches were conducted in PubMed, Web of Science, and EMBASE for randomised controlled trials that evaluated the influence of exercise on fall-induced injuries in older people.

**Results:**

Twenty-five trials met the inclusion criteria. Exercise significantly reduced the risk of fall-related injuries in older adults, risk ratio (RR) 0.879 [95% confidence interval (CI) 0.832–0.928]. Among the injuries, events needing medical care or resulting fractures were also decreased by exercise intervention, with RR 0.681 (0.562–0.825) and 0.561 (0.366–0.860), respectively. When analysis was stratified by participant characteristics and exercise protocols, we found that participants at high risk of falling, or with osteoporosis, were sensitive to exercise intervention. Combined exercise protocols and balance training were the most effective exercise types in reducing fall-related injuries. Exercise-associated beneficial effects were even significant in very old people (≥80 years) and across the duration of interventions (< 6 months, 6 to 12 months and ≥ 12 months). Exercise only generated a very low injury rate per participant year (0.002, 95% CI 0–0.05) and showed relatively good compliance of exercise (as reported in the included papers) (78.5, 95% CI 72.8–84.2%).

**Conclusions:**

Exercise is effective in preventing fall-induced injuries across a variety of baseline participant characteristics and exercise protocols. Exercise was associated with a low injury rate and had a good compliance, suggesting it is a feasible approach to managing fall-related injuries.

## Background

Fall-related injuries are one major source of public health problems among older people. The occurrence of fall-induced injuries frequently causes a high economic cost [1] and increases long term pain for patients [2]. More importantly, some types of serious fall-related injuries, such as fracture, are also the main source of morbidity and mortality. It was reported that 50% of survivors after fracture failed to regain their former levels of autonomy and mobility [3], and the mortality rate during the first year after hip fracture was between 8.4 and 36.0% [4]. The current evidence highlights the need for preventive strategies to decrease the incidence of fall-related injuries in older populations.

In the broad fields of preventive strategies, exercise is an essential approach that can help individuals to maintain or restore muscle strength, balance and posture control, bone mass, and performance of activities of daily life, and subsequently reduce the risk of fall and fall-related injuries [5–7]. Recently, a Cochrane review provided pooled evidence for the effects of exercise on fall reduction and confirmed that exercise generated beneficial effects on the prevention of fall-induced injuries in dwelling community older people [8]. There were two other meta-analyses which also reported exercise-associated beneficial effects on reduction of fall-related injuries [9, 10]. Although the effect of exercise on the risk of fall-related injuries has been explored, considerable questions remain unclear. Firstly, it remains unclear whether the effects of exercise on fall-induced injuries differ in a variety of health conditions (*eg*, osteoporosis, high risk of falling, and stroke). Secondly, it is still unknown what types of exercise interventions are effective in reducing fall-related injuries. The studies included in the meta-analyses [9, 10] frequently conducted a diversity of exercise programmes, and divergent intervention protocols might lead to different treatment effects. However, previous meta-analyses [9, 10] only determined a summary intervention effect on fall-related injuries, with the analysis neither stratified by different populations nor exercise protocols. In addition, one major concern of participants is the safety of exercise, but it has not been evaluated yet. Given those important issues, we performed a systematic review and conducted meta-analysis to determine the effect of exercise on fall-related injuries in various participant characteristics and with divergent intervention protocols. We also examined the safety and compliance of exercise for performance.

## Methods

### Search strategy and inclusion criteria

This systematic review and meta-analysis adhered to the Preferred Reporting Items for Systematic Reviews and Meta-Analyses (PRISMA) guidelines [11]. Electronic database searches were conducted in PubMed, EMBASE, and Web of Science up to Feb 1, 2019. Two authors (WB and XC) carried out the search processes according to the predetermined search strategy which were listed in Additional file 1: Text S1. No language restriction was applied for the electronic database search. We also checked reference lists of articles included and conducted a forward search.

The included studies should meet the following criteria: (1) randomized controlled trials (RCTs) that investigated the effects of exercise on fall-related injuries. Exercise conducted in trials was to improve physical functions, such as muscle strength, balance, and joint flexibility, which were expected to reduce fall risk and subsequent injuries; (2) studies compared exercise intervention against a control group, such as no intervention, attention, or shame exercise (such as light physical activities), which was expected to have no effects on physical functions and fall risk; studies conducting cointerventions, such as usual care (control) and usual care plus physical activities (exercise intervention) [12], were also eligible if the main purpose of the study was to investigate the role of exercise and the sole difference between the intervention and control was the exercise training; (3) participants aged 60 years and over.

### Outcome measures

The primary outcome of this study was fall-related injuries (injurious caused by a fall, *eg,* fall with wound, head trauma, medical care, fracture, or hospitalization, according to original investigators); and injuries resulting in fracture (a fall that resulted in fracture) and needing medical care (a fall that required medical attention, *eg,* attended hospital emergency department or required general practitioner consultation) were the secondary outcomes.

### Study selection and data collection

Two authors (WB and XC) performed title/abstract screening independently. After that, the full-text of potentially eligible studies was accessed by two authors (WB and XC) for final determination of eligibility. In the case of disagreement, consensus was frequently achieved by discussion between the authors (RZ, XC, and WB). We extracted the following information: sample size, participant age, countries, study design, exercise interventions (category, intensity, frequency, and duration), participant health conditions (osteoporosis, history of falls, history of fractures, etc.), attrition, exercise compliance, exercise-related injuries, and the number or rate of fall-related injuries. If data were not reported in original trials, we contacted corresponding authors to obtain the data.

### Risk of Bias assessment

Risk of bias assessment for each included study followed the recommendations of the Cochrane Collaboration [13] and made a small adaption for exercise intervention studies. We made such adaption due to the fact that baseline imbalance (*eg,* the number of participants, age, and body weight of participants) that are strongly related to outcome measures can cause bias in the intervention effect estimate. Sometimes, such imbalance is small but highly significant and suggests failure of randomization [14, 15]. For each trial, pairs of members of the review team (WB and XC) reported the following key domains: sequence generation; allocation concealment; blinding; incomplete outcome data; and “other bias” (baseline balance of intervention and control groups). Each domain was judged to be low, unclear, or high risk of bias. The final assessment for all studies was presented in a “risk of bias” table.

### Statistical analysis

Intervention effects were reported as risk ratio (RR) [16]. If only hazard ratio (HR) was presented, we assumed HR as RR for combing the effects estimates. If raw data were presented, we calculated RR using the ‘csi’ command in STATA [8]. Generic inverse variance method was used for pooling effects estimates because this method allows both adjusted and unadjusted data used in combining intervention effects. Firstly, we evaluated the overall summary RR and 95% confidence interval (CI) for fall-related injuries, fractures, and injuries needing medical care. And then, we re-analyzed treatment effects on fall-related injuries grouped by baseline participant health conditions, including osteoporosis, high-risk falling, and history of diseases (stroke). The intervention effects were also re-estimated by grouping exercise categories (combined exercise, balance training, and walking exercise), intervention duration (< 6 months, 6 months to < 12 months, ≥12 months), and participant age (< 80 years and ≥ 80 years), respectively. We evaluated the rate of intervention-related injuries for the assessment of the safety of exercise. If the rate of exercise-related injuries was not presented in included studies, we calculated it by comparing the total number of injuries during training with the actual length of time monitored for participants contributing data (person-years). We also pooled the data on the compliance of exercise from the included studies.

We used the Chi^2^ test (with a significance level at *p* < 0.10) and I^2^ (low: 0–30%; moderate: > 30–60%; high: > 60%) to assess between-study heterogeneity. The test for the overall effects (z score) was regarded as significant at *p* < 0.05. STATA version 15 software (*StataCorp LP, College Station, TX, USA*) was used to perform the meta-analysis.

### Sensitivity and publication Bias analyses

We carried out sensitivity analysis to explore the impact of risk of bias on effects estimates. Trials with at least one domain scored high risk or marked unclear risk were removed from the analysis. Sensitivity analysis was performed for fall-induced injuries, fractures, and injuries required medical help. To determine the risk of publication bias, we constructed funnel plots with treatment effects (RR) estimated from individual studies against a measure of study size (standard error of log RR). We also examined the likelihood of the presence of small-study effects (Begg’s test) for fall-induced injuries, fractures, and injuries required medical care.

## Results

### Characteristics of included studies

**Fig. 1:**
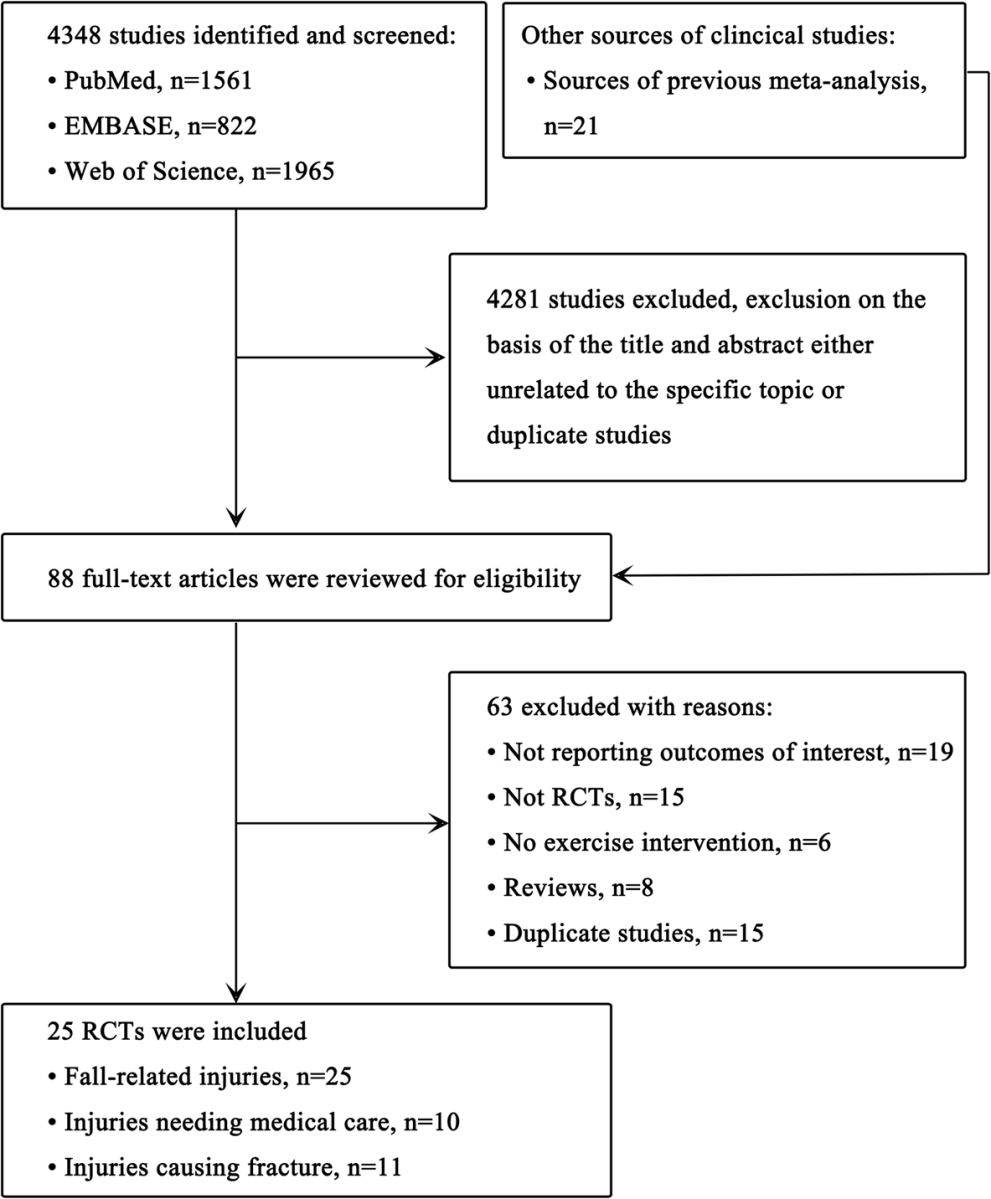
Flow chart for study selection

We identified and screened 4348 abstracts, of which 4281 were excluded because they were either unrelated to the topic or duplicate studies. We also identified 21 trials from other sources. Totally, 88 full-text articles were retrieved and reviewed for eligibility. Finally, 25 studies met the inclusion criteria, of which 25 reported fall-related injuries, 11 examined falls resulting fractures, and 10 determined injuries needing medical help (Fig.1). In total, 7076 participants were included, of which 3734 received exercise interventions and 3342 complied with the requirements for controls. The health conditions of participants comprised osteoporosis (exercise vs control: 2756 vs 2400), high risk of falling (923 vs 887), and stroke survivors (55 vs 55). The included studies were delivered both in community settings [17–31] and in institutions [12, 32–41]. The studies were carried out in US, UK, Germany, Australia, Sweden, New Zealand, France, China, Japan, Finland, Switzerland, and Netherlands (Table 1).

**Table 1:** Characteristics of included studies for fall-induced injuries

Authors	Study design	Ages (exercise, control)	Participants	Exercise intervention	CP	Control	Cases (exercise, control)
Barnett, 2003 [32], Australia	RCT; study duration: 1 yr	74.4 ± 4.9, 75.4 ± 6.0	163 older people	1 h weekly of structured exercise, including balance, coordination, aerobic capacity and muscle strength training, plus home exercise	62.2%	Usual lifestyle	22, 28
Bischoff-Ferrari, 2010 [12], Switzerland	RCT; study duration: 12 mos	83.4 ± 7.2, 85.1 ± 6.5	173 patients with acute hip fracture	Daily 60 mins of balance and strength training during acute care, continuing the programme at home after discharge	69.0%	Usual care	9, 16
Campbell, 1997 [17], New Zealand	RCT; training for 1 yr, following-up for 2 yrs	83.4 ± 2.7, 84.3 ± 3.3	233 older women	30 mins of home-based programme of strength and balance retraining exercises, plus walking, 3 times per wk	NS	Usual activity levels	27, 43
El-Khoury, 2015 [19], France	RCT; study duration: 2 yrs	79.8 ± 2.8, 79.6 ± 2.8	706 older women	Weekly supervised progressive balance training, supplemented by six weekly individually prescribed home exercises	NS	No intervention	305, 397
Elley, 2008 [20], New Zealand	RCT; study duration: 1 yr	80.4 ± 4.8, 81.1 ± 5.3	312 older people	Daily strength and balance exercise	NS	Social visits	170, 156
Fitzharris, 2010 [21], Germany	RCT; study duration: 3.8 mos	76.1	1090 older people	Weekly strength and balance exercise class of 1 h for 15 weeks, supplemented by daily home exercises.	NS	No intervention	358, 446
Freiberger, 2012 [22], Germany	RCT; training for 12 mos and following-up for 24 mos	76.1 ± 4.1	280 older people fallen in the past 6 months	1 h of strength and balance training, or plus strength training and balance exercises, or plus endurance training, 2 times per wk	84%	No intervention	26, 35
Haines, 2009 [23], Australia	RCT; study duration: 6 mos	80.9 ± 8.9, 80.5 ± 6.5	53 adults with gait instability	Exercise programme combining lower limb strength and balance exercises	NS	Usual care	15, 32
Iliffe, 2014 [24], UK	RCT; training for 6 mos, following-up for 1 yr	72.9 ± 6.1&72.8 ± 5.8, 73.1 ± 6.2	1256 older people	Group exercise: 1 h of strength training, plus 2 times of walking per wk.; home exercise: 3 times per wk. of muscle strengthening and balance exercises plus 2 times of walking	NS	Usual lifestyle	111, 88
Iwamoto, 2009 [33], Japan	RCT; study duration: 5 mos	74.6 ± 5.6, 78.2 ± 5.6	68 older people	30 mins of calisthenics, body balance training, muscle power training, and walking ability training, 3 times per wk	100%	No intervention	0, 4
Kim, 2014 [25], Japan	RCT; training for 3 mos, following-up for 1 yr	77.8 ± 4.2, 78.0 ± 4.2	105 older adults with a fall history	60 mins of muscle strength and balance training, 2 times per wk	75.3%	No intervention	8, 13
Li, 2005 [34], US	RCT; training for 26 wks, following-up for 1 yr	76.9 ± 4.7, 78.0 ± 5.1	256 older people	1 h of Tai Chi exercise, 3 times per wk	80.0%	No intervention	7, 17
Luukinen, 2007 [26], Finland	RCT; study duration: 2 yrs	88.0 ± 3.0, 88.0 ± 3.0	484 older people with a fall history	5–15 repetitions of daily home exercise or group exercise, including walking exercises or self-care exercises	NS	No intervention	39, 41
MacRae, 1994 [35], US	RCT; study duration: 12 mos	72.4 ± 0.9, 70.0 ± 0.9	80 older people	1 h of strength and balance exercise, 3 times per wk	NS	Safety Education	0, 3
Means, 2005 [27], US	RCT; training for 6 wks, following-up for 6 mos	73.5	338 older people	90 mins of active stretching, postural control, endurance walking, and repetitive muscle co-ordination exercises	NS	No intervention	15, 21
Pang, 2018 [28], China	RCT; 8-wk training and 6-mon follow-up	68.1 ± 9.0 vs 69.6 ± 10.8	84 stroke survivors	3 weekly 60-mins of balance and dynamic mobility training programme	93	Sham exercise	1, 6
Patil 2015 [29], Finland	RCT; study duration: 2 yrs	74.4 ± 2.9, 74.0 ± 3.1	409 older women with a history of falls	Twice weekly balance, weight bearing, strengthening, and functional exercises for the first 12 mos, and once weekly group and home exercise for remaining 12 mos	73% & 66%	Usual physical activity	115, 111
Reinsch, 1992 [36], US	RCT; study duration: 1 yr	73.3 ± 7.9, 75.9 ± 7.3	230 older people	1 h of stand-up/step-up exercises to improve strength and balance, 3 times per wk	NS	No intervention	11, 12
Robertson, 2001 [37], New Zealand	RCT; study duration: 1 yr	80.8 ± 3.8, 81.1 ± 4.5	240 older people	Muscle strengthening and balance exercise, 3 times a wk., plus a walking programme, 2 times a wk., training for 12 mos	71.0%	Usual care	42, 49
Rosendahl, 2008 [38], Sweden	RCT; training for 3 mos, following-up for 6 mos	85.3 ± 6.1, 84.2 ± 6.8	191 older people	29 sessions of progressively high intensity weight-bearing exercise to improve lower-limb strength, balance and gait ability	NS	The control activity program	64, 81
Schnelle, 2003 [39], US	RCT; study duration: 8 mos	87.3 ± 8, 88.6 ± 6.7	190 older people	5 sessions of walking or repeating sit-to-stand up, 5 days per wk	NS	Usual care	13, 21
Smulders, 2010 [40], Netherlands	RCT; training for 3 mos, following-up for 1 yr	70.5 ± 5.0, 71.6 ± 4.4	96 older people with fall history	Weight-bearing and walking exercises, and gait correction and fall prevention training programmes	92.8%	Usual lifestyle	20, 33
Taylor-Piliae, 2014 [30], US	RCT; study duration: 12 wks	71.5 ± 10.3, 68.2 ± 10.3	145 survivors of stroke	1 h of Taichi exercise, 3 times per wk	82.0%	Usual care	9, 13
Uusi-Rasi, 2015 [31], Finland	RCT; Study duration: 2 yrs	74.8 ± 2.9, 73.8 ± 3.1	409 women with a history of fall	Balance challenging, weight bearing, strengthening, and functional exercises, plus home-training program	72.8%	Take placebo	12, 26
von Stengel, 2009 [41], Germany	RCT; study duration: 18 mos	68.6 ± 3.0, 68.1 ± 2.7	151 postmenopausal women	Resistance training, plus impact and aerobic weight-bearing exercises, 4 days per wk	75.0%	Light physical exercises	17, 24

*yr(s)* year(s); *mo(s)* month(s); *wk(s)* week(s); *h(s)* hour(s); *min(s)* minute(s); *RM* repetition; *NS* no statement; *reps* repetitions; *CP* compliance

Three types of exercise programmes were identified in our meta-analysis, with 21 studies involving combined exercise (*ie*, multiple categories of ProFaNE taxonomy), 3 conducting balance training, and 1 performing walking exercise (*ie*, general physical activity of ProFaNE taxonomy). Combined exercise protocols included several distinct types of exercise (strength training, balance exercise, and aerobic activities, etc.) incorporated into one training class to augment the beneficial effects of exercise on bone and muscle [42–44].

Exercise interventions were delivered in centers (11 studies), homes (5 studies), or the combination of centers and homes (9 studies). Exercise delivered in centers frequently involved detailed guidelines, weekly or monthly exercise protocol adjustment, and constant supervision. When exercise was performed at home, participants usually received periodical visits and professional instructions ensuring correct performance and better adherence. Home-based exercise was sometimes performed as supplementatary programme to grouped-based training, or as an extension programme after finishing center-based intervention. Other detailed information about the included studies is listed in the Table 1.

### Meta-analysis

**Fig. 2:**
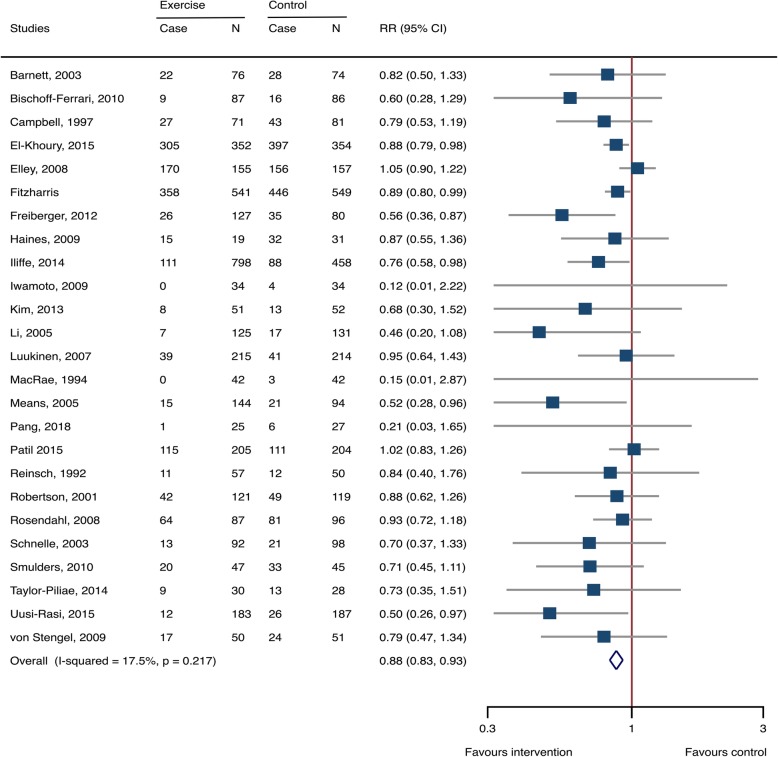
Exercise-associated treatment effects on fall-related injuries. *RR*: risk ratio; *CI*: confidence interval; *N*: number

**Fig. 3:**
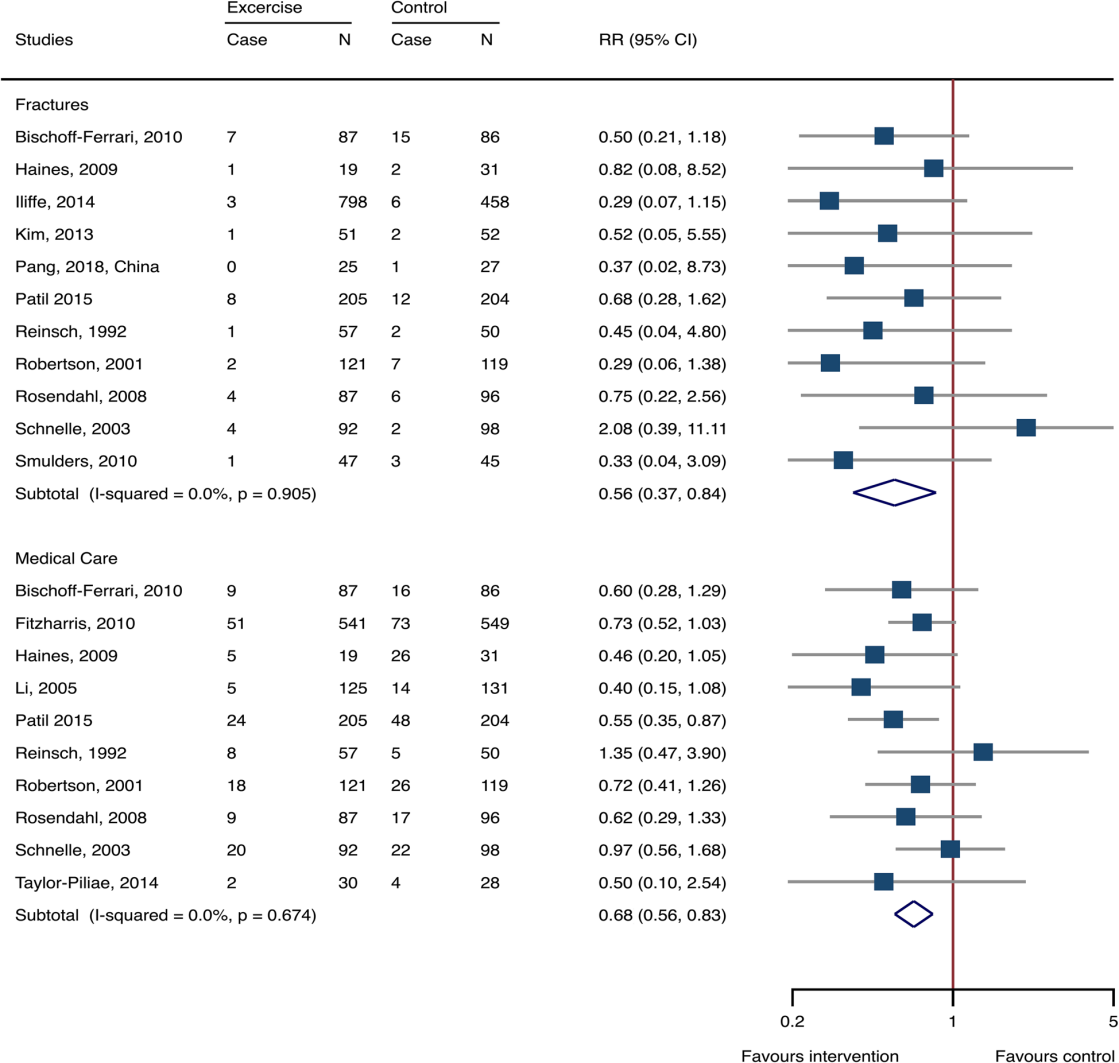
Exercise-associated treatment effects on injuries needing medical help and fractures. *RR*: risk ratio; *CI*: confidence interval; *N*: number

Fixed-effects methods were used for calculating effects sizes (Additional file 1: Table S1). Exercise significantly reduced the risk of fall-related injuries in older adults, with RR 0.879 (95% CI 0.832–0.928, 25 studies, 7076 participants, I^2^ = 17.5%) (Fig. 2). Among the fall-induced injuries, the events that needed medical care or caused fractures were also pronouncedly decreased by exercise intervention, with RR 0.681 (0.562–0.825, 10 studies, 2756 participants, I^2^ = 0%) and 0.561 (0.366–0.860, 11 studies, 2855 participants, I^2^ = 0%), respectively (Fig. 3).

**Fig. 4:**
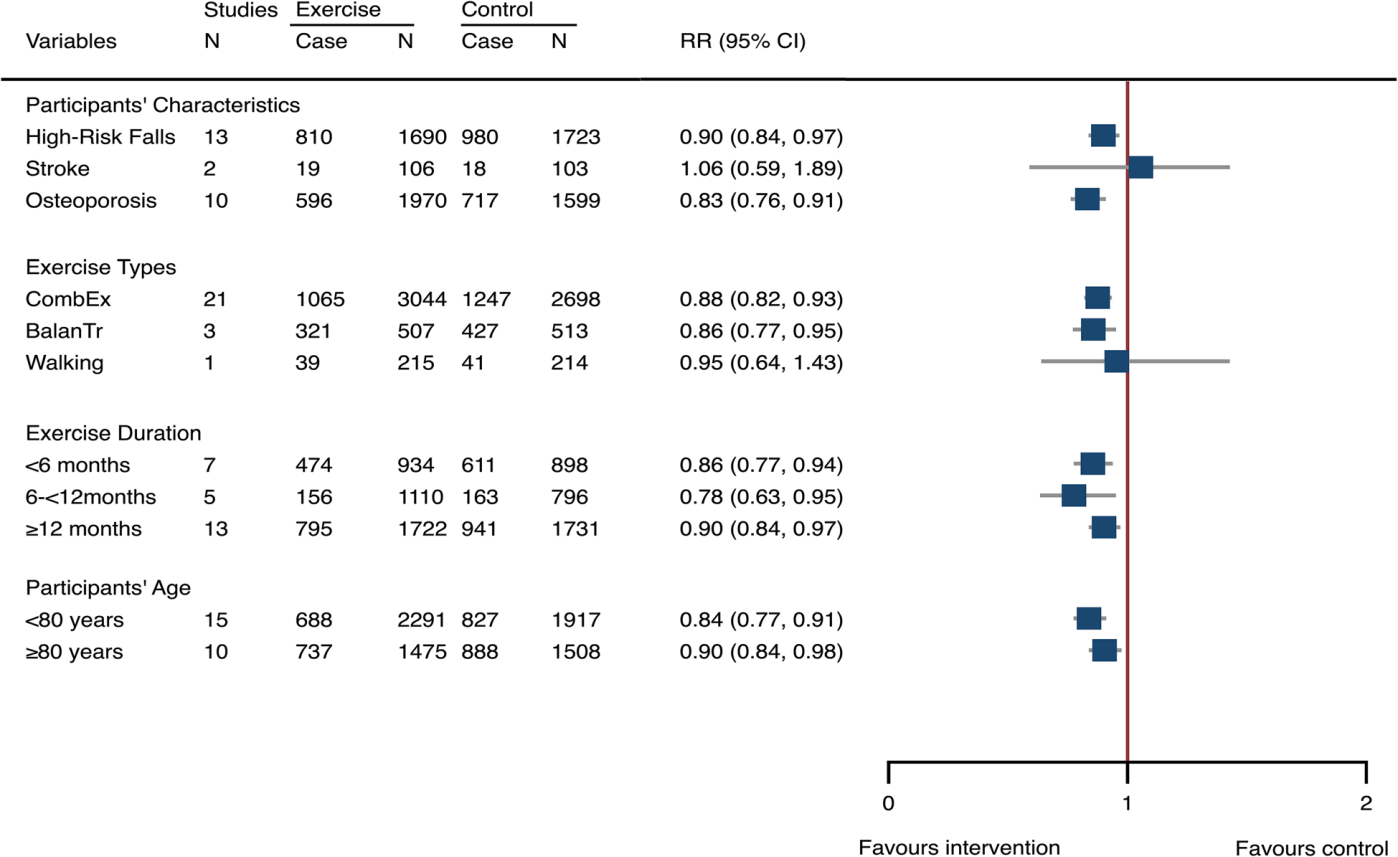
Exercise-associated treatment effects on fall-related injuries stratified by participants’ characteristics, exercise types, and exercise duration, and age. *CombEx*: combined exercise; *BalanTr*: balance training; *RR*: risk ratio; *CI*: confidence interval; *N*: number

We carried out meta-analyses by participant characteristics and exercise protocols to determine whether the treatment effects were affected by the variety of participant health conditions, exercise types, and training durations. Findings indicated that participants at high risk of falling (RR 0.899, 95% CI 0.837–0.966, 13 studies, 3413 participants, I^2^ = 30.5%) or with osteoporosis (0.832, 0.762–0.909, 10 studies, 3569 participants, I^2^ = 13.4%) were sensitive to exercise intervention, but stroke survivors did not show beneficial effects from exercise training (1.056, 0.589–1.895, 2 studies, 209 participants, I^2^ = 19.1%) (Fig. 4). The combined exercise protocols (0.875, 0.819–0.934, 21 studies, 5732 participants, I^2^ = 21.0%) and balance training (0.857, 0.771–0.952, 3 studies, 1020 participants, I^2^ = 28.6%) were effective in reducing fall-related injuries whereas walking exercise did not generate significant outcomes. Additionally, exercise yielded pronounced treatment effects for reducing fall-related injuries in all age ranges of older people (< 80 years and ≥ 80 years) and across different intervention durations (from < 6 months and 6 to < 12 months to ≥12 months), (Fig. 4).

To examine the safety and compliance of exercise we pooled the data of injuries during exercise training and the compliance of exercise throughout the training sessions from the included studies. Thirteen and five studies reported the data on compliance of exercise and training-induced injuries, respectively; it was suggested that exercise training only generated a very low injury rate per participant year (0.002, 95% CI 0–0.05, 4 studies, 604 training older people) and showed a relatively good compliance of exercise (as reported in the included papers) (78.5, 95% CI 72.8–84.2%, 13 studies, 1161 exercising participants).

### Study quality, sensitivity, and publication bias analyses

Of the 25 included trials, 13 were ranked low risk of bias in any domain; 4 and 1 scored high risk of bias in one and two domains. However, there was 7 studies that marked unclear in at least one domain, which prevented us from making a clear assessment (Table 2).

**Table 2:** Study quality assessment

Authors	Sequence generation	Allocation concealment	Blinding^a^	Incomplete outcome data	Baseline comparability
Barnett, 2003 [32],	Unclear	Low	Low	Low	Low
Bischoff-Ferrari, 2010 [12]	Low	Low	Low	Low	Low
Campbell, 1997 [17]	Low	Low	Unclear	High	Low
El-Khoury, 2015 [19]	Low	Low	Low	Low	Low
Elley, 2008 [20]	Low	Low	Low	Low	Low
Fitzharris, 2010 [21]	Low	Low	Low	Unclear	Low
Freiberger, 2012 [22]	Low	Low	Low	Low	Low
Haines, 2009 [23]	Low	Low	Low	Low	Low
Iliffe, 2014 [24]	Low	Low	High	Low	High
Iwamoto, 2009 [33]	Unclear	Unclear	Unclear	Low	High
Kim, 2014 [25]	Low	Low	Low	Low	Low
Li, 2005 [34]	Low	Unclear	Low	Low	Low
Luukinen, 2007 [26]	Low	Unclear	Low	Low	Low
MacRae, 1994 [35]	Unclear	Low	Low	Low	Low
Means, 2005 [27]	Low	Unclear	Unclear	Low	High
Pang, 2018 [28],	Low	Low	Low	Low	Low
Patil 2015 [29]	Unclear	Unclear	Unclear	Low	Low
Reinsch, 1992 [36]	Unclear	Unclear	Unclear	Low	Unclear
Robertson, 2001 [37]	Low	Low	Low	Low	Low
Rosendahl, 2008 [38]	Low	Low	Low	Low	Low
Schnelle, 2003 [39]	Low	Low	Low	Low	Low
Smulders, 2010 [40]	Low	Unclear	Low	High	Low
Taylor-Piliae, 2014 [30]	Low	Low	Low	Low	Low
Uusi-Rasi, 2015 [31]	Low	Low	Low	Low	Low
von Stengel, 2009 [41]	Low	Low	Low	Low	Low

Note: ^a^: Blinding for assessor due to the fact that exercise intervention study impossibly blinds for exercisers

To conduct sensitivity analysis, we first removed the trials with at least one domain scored high risk of bias. It was suggested that the intervention effects were still significant for fall-induced injuries (RR 0.894, 95% CI 0.845–0.947, 21 studies, 5311 participants, I^2^ = 14.8%), fractures (0.617, 0.390–0.976, 9 studies, 1507 participants, I^2^ = 0%) and injuries needing medical help (0.681, 0.562–0.825, 10 studies, 2756 participants, I^2^ = 0%) (Additional file 1: Table S1). We then further removed studies with the domains marked at least one unclear risk of bias; the treatment effects remained significant for fall-induced injuries (0.890, 0.826–0.959, 13 studies, 2745 participants, I^2^ = 25.0%) and events needing medical help (0.699, 0.523–0.953, 6 studies, 894 participants, I^2^ = 0%), but not for fractures (0.605, 0.348–1.052, 7 studies, 991 participants, I^2^ = 0%) (Additional file 1: Table S1). It indicated that though exercise generated a fracture reduction rate by 39.5%, the evidence of intervention effects was not certain.

Additionally, we combined both the funnel plots and small-study tests to examine the publication bias. The visual inspection of the funnel plots for fall-related injuries suggested there existed asymmetry (Additional file 1: Figure S1) and Begg’s test showed evidence for the presence of small-study effects (*p* = 0.047) (Additional file 1: Table S1). We then explored if the trials showing small-study effects affected summary effects estimates. After removing three trials [28, 33, 35] with obvious small-study effects, it resulted in a significant reduction of Begg’s test value (*p* = 0.063), and the intervention effects were still significant (0.881, 0.834–0.931). It was suggested though there existed in small-study effects, it did not affect the results much. The visual inspection of the funnel plots for fractures also suggested there seemed to exist asymmetry (Additional file 1: Figure S2), but there had weak evidence for the presence of small-study effects (Begg’s tests: *p* = 0.876) (Additional file 1: Table S1). The plots for falls needing medical care were relatively symmetry (Additional file 1: Figure S3), and Begg’s tests (*p* = 0.592) suggested there had weak evidence for the appearance of small-study effects (Additional file 1: Table S1).

## Discussion

Exercise was an effective approach to the prevention of fall-induced injuries (12% reduction), as well as injuries causing medical care (32% decrease) and fractures (44% reduction). The effect of exercise on prevention of fall-related injuries differed by a variety of participant characteristics and exercise protocols. Furthermore, exercise only induced a low injurious rate and had a good compliance, suggesting it is a feasible approach for older people to manage injurious falls. The heterogeneity between studies was relatively low; the sensitivity analysis suggested our results were robust for fall-related injuries and injuries needing medical care.

Our findings suggested that exercise-associated beneficial effects for prevention of fall-induced injuries were consistent in older people (aged from < 80 years to ≥80 years) and were also significant for different intervention durations (< 6 months, 6–< 12 months, and ≥ 12 months). Though previous meta-analyses [7, 9] also reported a beneficial effect on fall-related injuries after exercise intervention, they failed to determine whether the age of participants and the duration of interventions would affect the treatment effects. Our findings have clinical significance because the evidence proves exercise to be an effective strategy for management of fall-related injuries even for adults with advanced ages and interventions with relative short time. Recently, several evidence-based studies reviewed approach to the prevention of falls and related injuries, and confirmed exercise was a promising strategy for preventing fall-related injuries in older people [45, 46].

Generally, the risk factors contributing to falls and fall-related injuries are similar, including aging-related frailty, decrease of muscle strength, impaired balance function and posture control, and decreased performance of activities of daily life, most of which are modifiable [8, 10, 47, 48]. Our results suggested that, among the included studies that targeted on counteracting those adverse factors, combined and balance exercise programmes were most effective. By incorporating a variety of distinct types of exercise (such as resistance training, balance challenging, aerobic exercise and impact exercise), combined exercise protocols frequently generate multiple benefits in modifying risk factors causing falls and fall-induced injuries. Karinkanta et al [5] argued that not all forms of exercise were equally effective in fall prevention, and the most important components of exercise were combined exercise protocols. A Cochrane review [8] on intervention of preventing falls in community-dwelling older people also suggested that exercise which contained at least two types of exercise reduced both the risk of falls and fall rates.

Impairment of balance is recognized as the most frequent and sensitive risk factors for predisposing falls and subsequent injuries. Multiple risk factors, such as ageing, diseases (i.e. stroke and Parkinson’s Disease), immobilization and decreased physical activities, potentially decrease the individual’s ability of balance and posture control. Compelling evidence has confirmed that balance exercise is effective in correcting those risk factors, and shows beneficial changes in reducing falls and fall-induced injuries in older adults living in the community [8, 10, 47, 48].

Our results also demonstrated that walking did not help adults to manage the risk of fall-related injuries. However, due to the limited number of studies addressing this question further investigation is still necessary to ascertain our findings.

Our findings also suggested that for prevention of fall-related injuries participants at high risk of fall or with osteoporosis were mostly benefited by exercise intervention, but stroke survivors were not. The risk factors for fall and fall-related injuries that occur in osteoporotic and high-risk falling older person are also common in aged and frail older people, which are modifiable [8, 10, 47, 48]. But the situation in stroke survivors is more complicated. Those risk factors are more severe than in common older people and hard to modify easily. Therefore, Dean and colleagues [18] recommended that multiple prevention strategies, including exercise, education, home modifications, and medication, may be more beneficial in prevention of fall and subsequent injuries in stroke survivors.

In summary, exercise has the ability to reduce the risk of fall and fall-related injuries mainly by maintaining or restoring muscle strength, balance and posture control, bone mass, and performance of activities of daily life [5–7].

Data based on the included RCTs showed that exercise only generated a relative low injury rate. The major included studies adopted a protocol of combined exercise interventions and most of them frequently had no or only one exercise-induced injury. One population-based study [19] conducted a 24-month exercise programme with 352 exercising older participants and only reported 4 injuries during exercise classes. Furthermore, even for very old people exercise remained safe for practice. Robertson et al [37] enrolled a population of elderly adults aged ≥80 years and only one exercise-related injury occurred. However, only 5 studies reported injurious data during exercise and most included studies did not report this information, so we could not make a judgement on whether exercise-related injuries happed or not in those studies. Therefore, it is suggested that the exercise-related injuries should be recorded and reported, which will help to assess the safety of exercise for performance.

The compliance of exercise in most training protocols was relatively good. Freiberger et al [22] reported a percentage of exercise compliance about 84% during 12-month exercise intervention period. Uusi-Rasi and colleagues [31] found that participants with combined exercise programmes showed a relatively good compliance of exercise (73%). And several balance intervention studies [30, 34] also reported a very good compliance of exercise, 80 and 82%, respectively.

Our study was different from previous meta-analyses. Previous reviews [7–9], however, only determined the treatment effects on fall-induced injuries, fractures, or events needing medical care on a summary level disregarding the obvious differences in the participant characteristics and intervention protocols. We evaluated the different treatment effects between the participant health conditions, ages, exercise types, and intervention durations. Our findings demonstrated that exercise was a promising strategy for reducing fall-induced injuries in older people, especially for adults with osteoporosis or at high risk of falling, because it was not only effective, but safe and compliant for performance. Our findings have the potential to greatly benefit older people through the provision of recommendations on exercise intervention protocols based on various participant health status.

Generally, the heterogeneity between studies was relatively low. However, thought most individual studies had low risk of bias and only 5 studies showed high risk of bias, there remained several studies that did not provide the detailed information about the methodological profiles which might exist in some unclear risk of bias. Additionally, we used baseline imbalance as a domain to detect the risk of bias. Three studies scored high risk and one study marked unclear for baseline imbalance domain. Interestingly, studies with baseline imbalance domain either scored high or unclear risk frequently had high or unclear risk in other domains, which might imply baseline imbalance was potentially associated with the quality of study. The sensitivity analysis demonstrated that the certainty of evidence for intervention effects on fall-related injuries and injuries needing medical care were high, but for fractures were low. Additionally, a limited number of included studies for fractures and falls needing medical care might be not enough to make a decision on whether there really existed small-study effects. The major limitation of this study was that some groups of analyses (*eg*, stroke survivors and walking exercise) included few studies. In addition, the number of included studies that reported intervention-related injuries were also limited. Therefore, any elucidation of those results should be taken caution.

## Conclusion

Our meta-analysis has examined the efficacy and safety of exercise in preventing fall-related injuries across various participant characteristics and exercise programmes. The findings have clinical significance because exercise provides older peoples an alternative intervention strategy to prevent fall-induced injuries. However, for some special patients such as stroke survivors, future investigation is still needed due to the limited number of eligible studies included.

## Supplementary information

**Additional file 1: Text S1.** Search strategy in PubMed. **Table S1**. Summary, small-effects, and sensitivity analysis. **Figure S1.** Funnel plots of fall-related injuries. **Figure S2.** Funnel plots of fractures. **Figure S3.** Funnel plots of falls needing medical help.

## Data Availability

The datasets generated and analysed during the current study are available from the corresponding author on reasonable request.

## Abbreviations

CI: Confidence interval
HR: Hazard ratio
PRISMA: Preferred Reporting Items for Systematic Reviews and Meta-Analyses
RCTs: Randomized controlled trials
RR: Risk ratio
