# Supplementary material for: The efficacy and safety of exercise for prevention of fall-related injuries in older people with different health conditions, and differing intervention protocols: a meta-analysis of randomized controlled trials
Source: BMC Geriatr. 2019 Dec 3;19:341. doi: 10.1186/s12877-019-1359-9 (PMC6892137; doi:10.1186/s12877-019-1359-9)

**Additional file 1: Text S1:** Search strategy in PubMed

1 exercis*[Title/Abstract]

2 physical activit*[Title/Abstract]

3 resistance training[Title/Abstract]

4 strength training[Title/Abstract]

5 balance training[Title/Abstract]

6 walking[Title/Abstract]

7 running[Title/Abstract]

8 Tai Chi Quan[Title/Abstract]

9 (#1 OR #2 OR #3 OR #4 OR #5 OR #6 OR #7 OR #8)

10 fall*[Title/Abstract]

11 injur*

12 (#10 AND #11)

13 (#9 AND #12)

**Additional file 1: Table S1**: Summary, small-effects, and sensitivity analysis

|  | **Fall-related injuries** | **Fractures** | **Medical care** |
| --- | --- | --- | --- |
| **Summary analysis** | | | |
| Studies (n) | 25 | 11 | 10 |
| *Participants (n)* | 7076 | 2855 | 2756 |
| *I^2^* | 17.5% | 0.0% | 0.0% |
| RR (95% CI) | 0.879 (0.832-0.928) | 0.561 (0.366-0.860) | 0.681 (0.562-0.825) |
| p value | <0.0001 | 0.008 | <0.0001 |
| **Small study analysis** | | | |
| Studies (n) | 25 | 11 | 10 |
| Begg's Test (p value) | 0.047 | 0.876 | 0.592 |
| **Sensitivity analysis I** | | | |
| Studies (n) | 21 | 9 | 10 |
| RR (95% CI) | 0.894 (0.845-0.947) | 0.617 (0.390-0.976) | 0.681 (0.562-0.825) |
| p value | <0.0001 | 0.039 | <0.0001 |
| **Sensitivity analysis II** | | | |
| Studies (n) | 13 | 7 | 6 |
| RR (95% CI) | 0.890 (0.826- 0.959) | 0.605 ( 0.348-1.052) | 0.699 (0.523-0.935) |
| p value | 0.002 | 0.075 | 0.0016 |

**Notes.** Sensitivity analysis I: sensitivity analysis carried out after removing the studies with high risk of bias in at least one domain. Sensitivity analysis II: sensitivity analysis carried out after removing studies with both high risk and unclear of bias in at least one domain.

**Additional file 1: Fig. S1:** Funnel plots of fall-related injuries


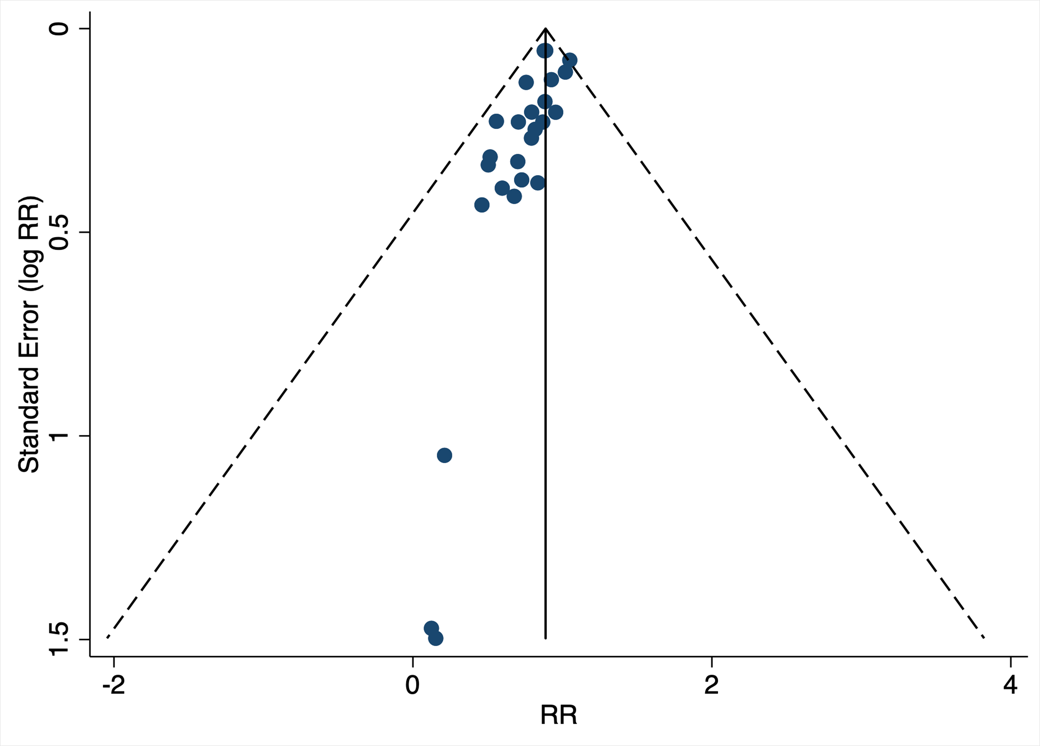


**Additional file 1: Fig. S2:** Funnel plots of fractures


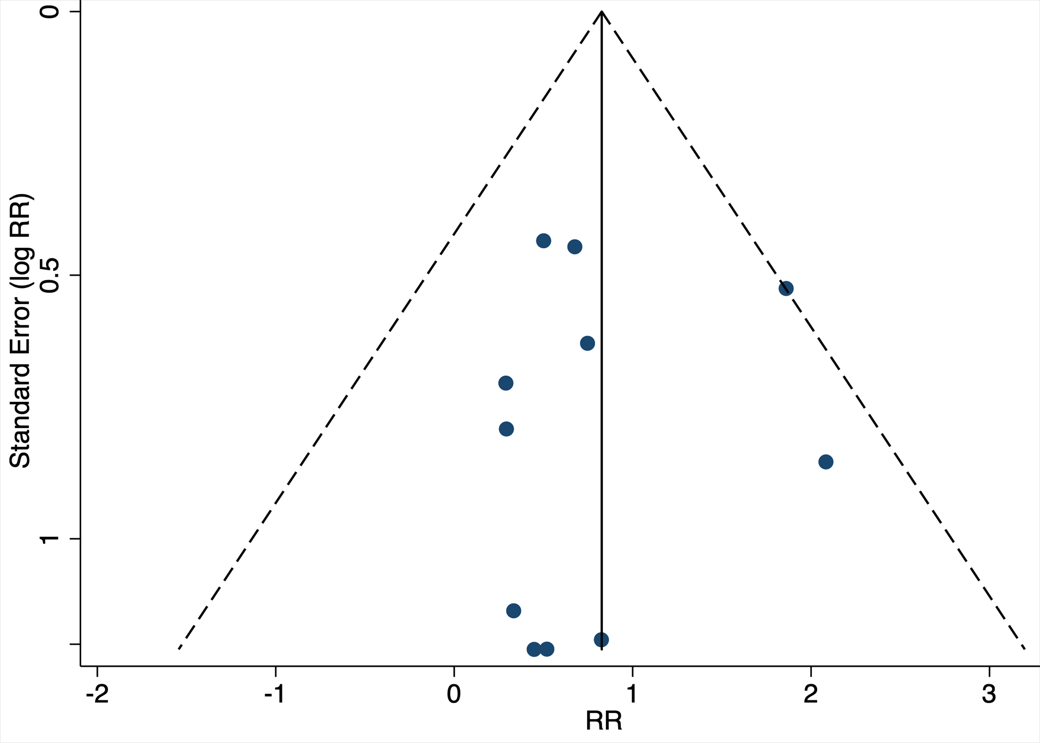


**Additional file 1: Fig. S3:** Funnel plots of falls needing medical help


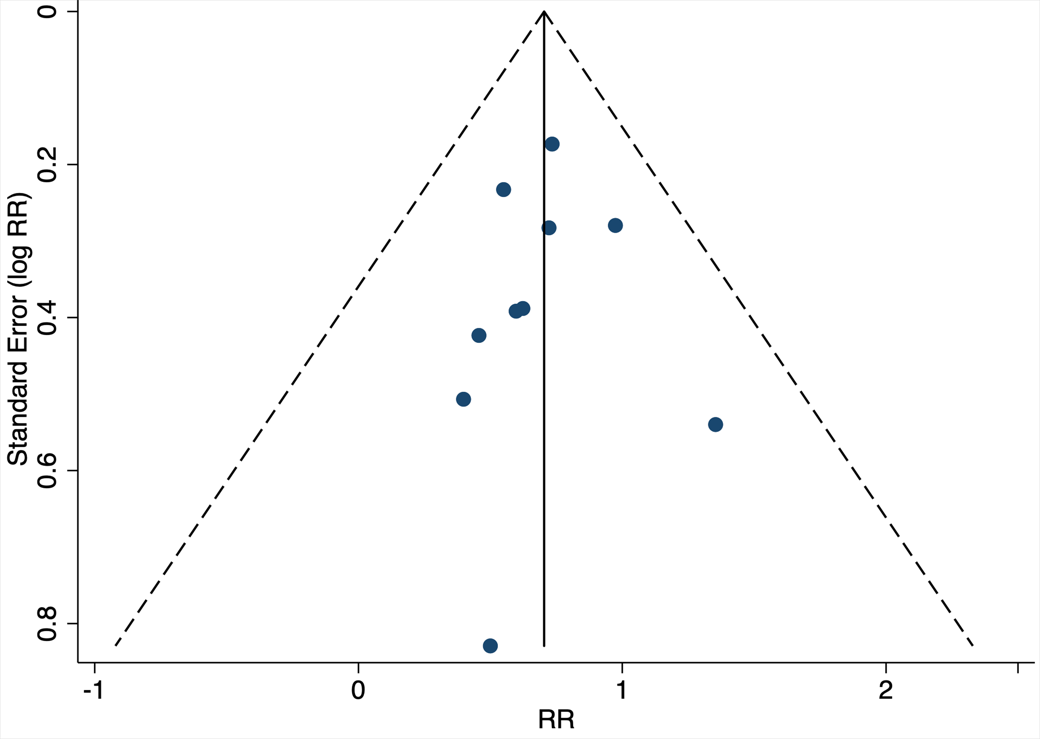

Supplement: Supplementary file 1 — Additional file 1: Text S1. Search strategy in PubMed. Table S1. Summary, small-effects, and sensitivity analysis. Figure S1. Funnel plots of fall-related injuries. Figure S2. Funnel plots of fractures. Figure S3. Funnel plots of falls needing medical help. [file 12877_2019_1359_MOESM1_ESM.docx]
